# Severe hypoglycemia symptoms, antecedent behaviors, immediate consequences and association with glycemia medication usage: Secondary analysis of the ACCORD clinical trial data

**DOI:** 10.1186/1472-6823-12-5

**Published:** 2012-05-30

**Authors:** Denise E Bonds, Michael E Miller, Jim Dudl, Mark Feinglos, Faramarz Ismail-Beigi, Saul Malozowski, Elizabeth Seaquist, Debra L Simmons, Ajay Sood

**Affiliations:** 1 National Heart Lung and Blood Institute, National Institute of Health, Bethesda, MD, USA; 2 Department of Biostatistical Sciences, Division of Public Health Sciences, Wake Forest University School of Medicine, Winston-Salem, NC, USA; 3 Care Management Institute, Kaiser Permanente, Oakland, CA, USA; 4 Department of Medicine, Duke University Medical Center, Durham, NC, USA; 5 Department of Medicine, Case Western Reserve University, Cleveland, OH, USA; 6 Division of Diabetes, Endocrinology and Metabolic Diseases, National Institute of Diabetes and Digestive and Kidney Diseases, Bethesda, MD, USA; 7 Division of Endocrinology and Diabetes, Department of Medicine, University of Minnesota Medical School, Minneapolis, MN, USA; 8 Division of Medicine, Endocrinology, Central Arkansas Veterans Healthcare System and University of Arkansas for Medical Sciences, Little Rock, AR, USA; 9 Division of Endocrinology, Department of Medicine, Case Western Reserve University, Cleveland, OH, USA

**Keywords:** Hypoglycemia, Type 2 diabetes

## Abstract

**Background:**

Hypoglycemia is a common complication of diabetes treatment. This paper describes symptoms, predecessors, consequences and medications associated with the first episode of severe hypoglycemia among ACCORD participants with type 2 diabetes, and compares these between intensive (Int: goal A1C <6.0%) and standard (Std, goal A1C 7–7.9%) glycemia intervention groups.

**Methods:**

Information about symptoms, antecedents, and consequences was collected at the time participants reported an episode of severe hypoglycemia. Data on medications prescribed during the clinical trial was used to determine the association of particular diabetes drug classes and severe hypoglycemia.

**Results:**

The most frequently reported symptoms in both glycemia group were weakness/fatigue (Int 29%; Std 30%) and sweating (Int 26%; Std 27%), followed by confusion/disorientation (Int 22%; Std 29%) and shakiness (Int 21%; Std 19%). Approximately half of all events were preceded by a variation in food intake (Int 48%; Std 58%). The most common consequences were confusion (Int 37%; Std 34%), loss of consciousness (Int 25%; Std 25%), and hospitalization (Int 18%; Std 24%). The highest rates of hypoglycemia were found among those participants treated with insulin only (Int 6.09/100 person yrs; Std 2.64/100 person yrs) while the lowest were among those prescribed oral agents only (Int 1.93/100 person yrs; Std 0.20/100 person yrs).

**Conclusions:**

Severe hypoglycemia episodes were frequently preceded by a change in food intake, making many episodes potentially preventable. Symptoms of confusion/disorientation and loss of consciousness were frequently seen. The highest rates of hypoglycemia were seen with prescription of insulin, either alone or in combination with other medications.

**Clinical Trial Registration:**

Number: NCT00000620

## Background

Hypoglycemia is a common complication associated with treatment of diabetes [1]. Often mild, in its more severe forms hypoglycemia can cause seizures, coma, and even death. Although hypoglycemia occurs more frequently in patients with type 1 as compared to type 2 diabetes [1], clinicians often see more hypoglycemia in patients with type 2 diabetes simply because of the high prevalence of disease. One study that used a restrictive definition of hypoglycemia (requiring medical assistance) found similar rates for both type 1 and type 2 diabetes [2]. Indeed, the rising number of patients with type 2 diabetes in the United States and the focus on tighter glucose control for these patients has resulted in an increase in the number of hypoglycemic [3] reports in this group.

The most common causes of hypoglycemia among patients with type 2 diabetes include the use of hypoglycemic medications [4], inadequate caloric intake, exercise, errors with use of medications, and intercurrent illnesses. A better understanding of the association of these various variables with hypoglycemia would assist in counseling patients to manage and prevent this complication. The Action to Control Cardiovascular Risk in Diabetes (ACCORD) study offers a unique opportunity to examine the immediate predecessors to severe hypoglycemic events. As part of routine safety monitoring of study participants, extensive reporting of the immediate predecessors and consequences of severe hypoglycemia were systematically captured. In addition, a comprehensive list of prescribed glycemia medications was kept.

The purpose of this paper is to describe the symptoms reported by ACCORD subjects who experienced severe hypoglycemia requiring medical assistance (HMA). Comparisons between glycemia intervention study groups (intensive strategy including a goal A1C < 6% versus standard strategy including goal A1C 7–7.9%) are made for predecessors and consequences to determine if there are differences by study group. Furthermore, comparisons between those with and without a reported episode of HMA are made for medication usage to determine if prescription of a particular class of medication or prescriptions of combination of medications is associated with severe hypoglycemia.

## Methods

The ACCORD Study is a double 2x2 factorial trial designed to test the effect of an intensive glucose control strategy defined as a goal hemoglobin A1C ≤ 6.0%, versus a standard control strategy (goal hemoglobin A1C between 7.0 and 7.9%), intensive blood pressure control strategy (SBP < 120 mmHg) versus standard control strategy (SBP < 140 mmHg), and a lipid treatment strategy that utilizes fenofibrate plus a statin, compared to one that uses a statin alone, on the composite outcome of myocardial infarction, stroke or cardiovascular death [5]. The intensive glycemia intervention was stopped early due to increased mortality in this group, and all participants were transitioned to the standard glycemia intervention for the remainder of the trial. This analysis is limited to the period of time prior to this transition. Main results have been previously reported [6-8], as has a full description of the methodology and the rationale for the trial [5,9]. All clinical sites obtain Institutional Review Board approval for the ACCORD Study.

### Study participants and design

Briefly, participants were eligible to enroll in ACCORD if they had type 2 diabetes, a hemoglobin A1C of 7.5-11%, and were either between the ages of 40 and 79 years with cardiovascular disease, or between the ages of 55 and 79 years with evidence of significant atherosclerosis, albuminuria, left ventricular hypertrophy, or two or more additional risk factors for cardiovascular disease (dyslipidemia, hypertension, current smoker, or obesity). Participants also needed to meet inclusion criteria for blood pressure and/or lipid trials. Participants were excluded if they had a history of frequent or recent serious hypoglycemic events, history of coma/seizure within the last 12 months, unwillingness to do home glucose monitoring or inject insulin, a body-mass index (the weight in kilograms divided by the square of the height in meters) of more than 45 kg/m^2^, a serum creatinine level of more than 1.5 mg per deciliter (133 μmol per liter), or other serious illness.

Participants were randomly assigned to intensive or standard glycemia intervention strategy. All participants received education and counseling about diabetes care. Supplies for glucose monitoring and glucose-lowering medications (from a study-supervised formulary of FDA-approved medications) were provided. Glucose lowering medications not on the ACCORD formulary could be prescribed to participants but were not provided free of cost to participants. Medications were utilized at the discretion of the study physician, with a suggested algorithm of order of use provided in the protocol. Use of medication class was not limited by study assignment. Intensive group participants were expected to be seen and medications adjusted every 2 months with at least one phone contact between visits while standard group participants were expected to be seen every 4 months (or more frequently as needed). Self-monitoring of blood glucose (SMBG) was advised for participants in both groups with more frequent monitoring recommended for intensive group participants and less frequent for standard group participants. Participating clinical sites included diabetes clinics, primary care clinics and dedicated research clinics. For this report, we used data for all follow-up contacts where ACCORD physicians actively managed the participant’s glycemia medications through February 5, 2008, the date that participants were notified that intensive glycemia participants were to be transitioned to standard glycemia therapy.

### Definition and reporting of hypoglycemia

Participants were asked at every visit if they had experienced episodes of low blood sugar. A full description of the review of such events, including the adjustment of therapeutic goals in response to severe hypoglycemia, has been previously reported [10]. The present report focuses on symptomatic, severe hypoglycemic events requiring medical assistance (hospitalization, visit to the emergency room, treatment by medical personnel including emergency medical technician either in a clinical setting or at home) which was defined in ACCORD as either a blood glucose less than 50 mg/dl (2.8 mmol/L) or symptoms that promptly resolved with oral carbohydrate, intravenous glucose, or glucagon. NOTE: Prior to March 2003, clinic sites were not required to document the blood glucose level of the participant during a hypoglycemic episode requiring medical assistance. Fifteen percent of the episodes used in the analyses in this paper do not have this documentation. Exclusion of events without this documentation does not qualitatively change the conclusions. (Data not shown.)

Following a report of a severe hypoglycemic episode by the participant, research staff inquired about the circumstances surrounding the episode. Standardized forms that utilized lists, check boxes and free text boxes were used to collect information. The time of the event (4 hour intervals check boxes) and whether the event had occurred while the participant was asleep was collected. Symptoms that the participant experienced prior to treatment of the hypoglycemia were ascertained and recorded. Symptom choices included both physical symptoms (e.g., shakiness, fast heartbeat) as well as psychological (e.g., anxiety) and neuroglycopenic (e.g., confusion). More than one symptom could be reported.

Activities and circumstances immediately antecedent to and consequences of the event were also obtained. Antecedents included changes in diet and exercise, changes in both glucose lowering medications and other medications, and intercurrent illnesses. Potential consequences included physiological responses (loss of consciousness, seizure), emergency treatment, and accident or injury. For both antecedents and consequences, more than one response was possible.

### Medications

Prescribed medications were recorded at each study visit. Oral glucose lowering medications were documented with both the dose and frequency prescribed at the time of the study visit. Insulin was noted on a separate management sheet. For both oral glucose- lowering medications and insulin, the type and dose of the medication reported by the participant at the beginning of the study visit and changes made in the regimen at the study visit were recorded. For this analysis, participants are assumed to be taking the prescribed medications at the time of the hypoglycemic episode if the medication was prescribed at the exit from the study visit just prior to the reported severe hypoglycemia event. Although the ACCORD protocol did not limit the type of glucose-lowering medication that could be used in the study, this analysis focuses on those medications most commonly used among the ACCORD participants. For analysis, medications are also combined based on mechanism of action (insulin sensitizer = TZD, metformin; basal insulin = NPH, lente, ultralente, glargine, insulin pump, detemir; bolus insulin = regular, aspart, lispro, glulisine; premixed = 70/30, 75/25, 50/50). Medication prescription was categorized for each period of follow-up in terms of use of: just oral agents alone, just insulin alone, oral agents and insulin combined, or no glycemia medications. Additionally, the number of different medications was categorized as 0 or 1, 2, or 3+ within each follow-up interval by calculating the number of the following medications prescribed: sulfonlyureas, biguanides, thiazolidinediones (TZD), alpha glucosidase inhibitors (AGIs), meglitinides, incretins, bolus insulin, basal insulin, and premixed insulin. For this analysis, we used all medication information until the time of the initial hypoglycemia event or until the final visit when participants had their glycemia medications actively managed by ACCORD physicians.

### Statistical analysis

All statistical analyses were conducted at the coordinating center with the use of SAS software, version 9.2 (SAS Institute). All analyses were conducted on the first reported hypoglycemic event requiring medical assistance (HMA). Symptoms reported at the time of the event and immediate antecedents to events are summarized as the number and percent of all initial events.

Use of glucose-lowering medications was summarized according to study group as the total person-years that participants were prescribed medications. Person-years were determined by using clinic reports of what was prescribed for the interval of time between each clinic visit. Rates of HMA per 100 person-years of medication use was obtained by taking the number of initial events that occurred while participants were prescribed a medication divided by the total number of person-years of use.

Hazard ratios (95% CI) relating the risk of HMA to prescription of medications were obtained within glycemia treatment groups using Cox proportional hazards (PH) regression models allowing for medication use as time-dependent covariates and controlling for the baseline covariates identified in Miller et al. [11] as predictive of future episodes of severe hypoglycemia. These baseline covariates included: age, gender, race, education, BMI, history of neuropathy/nerve problems, time since diagnosis of diabetes, A1C, albumin to creatinine ratio, serum creatinine, LDL-C, as well as factors used to stratify randomization (treatment groups within the BP and Lipid trials and the presence of clinical cardiovascular disease). Series of models that were fitted included baseline covariates plus each of the following as a single additional factor entered into the model: 1) each medication; 2) the oral/insulin medication category variables; 3) the number of medications. In addition to these models, we fitted models with all medications in the model and *a priori* selected the following interactions to investigate: oral agents and insulin, insulin and insulin sensitizers; metformin, sulfonylurea and TZDs; insulin, sulfonylurea and TZD.

## Results

A total of 732 first time severe hypoglycemic events were reported by ACCORD participants. Three times more events occurred among participants in the intensive group (N = 547) than those in the standard group (N = 185). There were no differences by study group in the percentage of participants with severe hypoglycemia who had documented blood glucose less than 50 mg/dl or who reported the occurrence of the event while asleep (see Table 1). Similarly, there were no difference in the reported symptoms by study group with the most frequent being symptoms of weakness/fatigue, sweating, and confusion/disorientation and shakiness. Episodes tended to occur less frequently at night than during the day. In addition, episodes among participants in the standard group tended to occur with increased frequency between 12 AM and 12 PM while participants in the intensive group had more episodes between 12 PM and 12 AM.

**Table 1:** Comparison by glycemia intervention group of the reported symptoms at the time of the hypoglycemia event

	**Intensive Glycemia N = 547% (n)**	**Standard Glycemia N = 185% (n)**	**P value**
Documented blood glucose <50 mg/dl	**63% (344)**	**63% (116)**	**0.96**
Event occurred while participant was asleep	**18% (101)**	**17% (31)**	**0.60**
Reported symptoms at the time of event			
Weakness/Fatigue	**29% (160)**	**30% (55)**	**0.90**
Sweating	**26% (144)**	**27% (50)**	**0.85**
Confused or Disoriented	**22% (123)**	**29% (53)**	**0.09**
Shakiness	**21% (116)**	**19% (36)**	**0.61**
Dizziness	**16% (86)**	**14% (25)**	**0.47**
Blurry Vision	**9% (49)**	**14% (25)**	**0.08**
Anxious	**10% (57)**	**8% (14)**	**0.26**
Irritable	**8% (44)**	**8% (14)**	**0.84**
Fast Heartbeat	**4% (22)**	**4% (7)**	**0.89**
Hungry	**5% (26)**	**2% (4)**	**0.12**
Headache	**3% (14)**	**2% (3)**	**0.46**
Time of Event			**0.08**
12:01 am - 4 am	**10% (40)**	**11% (15)**	
4:01 am - 8 am	**12% (47)**	**18% (26)**	
8:01 am - 12 pm	**23% (91)**	**26% (37)**	
12:01 pm - 4 pm	**21% (86)**	**20% (29)**	
4:01 pm - 8 pm	**20% (81)**	**19% (27)**	
8:01 pm - 12 am	**14% (58)**	**6% (9)**	

The most frequently listed antecedents to a severe hypoglycemic episode can be characterized as behavioral. Approximately half of all severe hypoglycemic episodes were preceded by a food related incident (either a delayed or missed meal or had less carbohydrate consumption than anticipated), with a higher percentage of participants in the standard group than in the intensive group reporting food related antecedents (see Table 2). Unexpected or more vigorous exercise than usual for the participant was also a relatively common antecedent but did not vary by study group. Incorrect medication dosage and intercurrent illness were relatively uncommon antecedents.

**Table 2:** **Comparison by glycemia intervention group of reported antecedents and consequences of severe hypoglycemia**^**(1)**^

	**Intensive Glycemia N = 478% (n)**	**Standard Glycemia N = 172% (n)**	**P value**
**Immediate antecedents to severe hypoglycemic event:**			
None	**14% (79)**	**11% (20)**	**0.21**
Food Related	**48% (263)**	**58% (107)**	**0.02**
Delayed or missed meal	**31% (167)**	**44% (81)**	**<.01**
Ate less carbohydrate than prescribed or usual at preceding meal or snack	**26% (144)**	**25% (47)**	**0.81**
Ingested alcohol	**3% (18)**	**2% (4)**	**0.44**
Exercised unexpectedly or more vigorously than usual	**15% (80)**	**12% (23)**	**0.46**
Took incorrect type and dose of insulin (e.g. aspart instead of glargine at hs)	**1% (8)**	**2% (4)**	**0.52**
Took more insulin than prescribed or usually administered	**5% (30)**	**7% (13)**	**0.44**
Took more sulfonylurea or meglitinide than prescribed	**1% (7)**	**2% (3)**	**0.73**
Started on new medication known to cause hypoglycemia or to mask symptoms	**2% (10)**	**8% (14)**	**<.01**
Experienced recent weight loss without appropriate adjustment of insulin dose	**3% (15)**	**3% (6)**	**0.72**
Has an intercurrent illness as a cause without an error in exercise/food/drugs	**4% (22)**	**3% (6)**	**0.63**
Cognitive impairment/decline	**6% (33)**	**11% (20)**	**0.03**
Other	**23% (124)**	**24% (45)**	**0.64**
**Immediate consequences of severe hypoglycemia event**			
None	**3% (17)**	**1% (1)**	**0.05**
ER visit or seen by EMT (without hospitalization)	**67% (364)**	**75% (138)**	**0.04**
Confusion/irrational behavior	**37% (203)**	**34% (62)**	**0.38**
Coma/loss of consciousness with no seizure	**25% (137)**	**25% (47)**	**0.92**
Hospitalization	**18% (99)**	**24% (44)**	**0.09**
Personal Accident or injury	**6% (33)**	**6% (12)**	**0.82**
Seizure	**2% (10)**	**3% (6)**	**0.26**
Injury to another individual	**1% (3)**	**1% (1)**	**0.99**
Other	**6% (35)**	**8% (14)**	**0.58**

^(1)^ Only collected on events occurring after August 2003.

Emergency room visits, treatment by EMTs and hospitalizations were all common but likely due to the definition of severe hypoglycemia used in this analysis. Approximately one third of the episodes were associated with confusion or irrational behavior (see Table 2), and one quarter resulted in a loss of consciousness. There were 45 reported personal accidents or injuries, including 3 reports of fractures, 5 participants with lacerations, and 5 motor vehicle accidents with no difference by study group.

Participants in both glycemia groups who were treated with only oral medications had a lower rate of hypoglycemic episodes than those on insulin alone or insulin plus oral agents (Table 3). The rate of hypoglycemia was highest in those on insulin alone in both groups, averaging about 6 episodes for every 100 person-years in the intensive group and almost 3 episodes per 100 person-years in the standard group. Those participants treated with insulin plus oral medication had a rate of hypoglycemia that was between those treated with only oral agents and those treated with insulin alone, 3.31 to 3.69 per 100 person years in the intensive and 1.60-1.85 per 100 person years in the standard group.

**Table 3:** **Association of hypoglycemic events with glycemia medication class**^**(1)**^

	**Intensive Glycemia**			**Standard Glycemia**		
**Medication class**	**Person yrs of use of medication or combination**	**# of HMA events associated medication use**	**# events per 100 Person yrs**	**Person yrs of use of medication or combination**	**# of HMA events associated medication use**	**# events per 100 Person yrs**
**All Cases**	17141.4	547	3.19	17709.9	185	1.04
**Combination of medication classes**						
**Oral medication only**^**(2)**^	5691.6	110	1.93	9202.7	18	0.20
**Metformin, TZD**^**(3)**^	10342.1	300	2.90	5179.3	46	0.89
**Metformin, Sulfonylurea, TZD**^**(4)**^	6073.3	165	2.72	3360.7	18	0.54
**Insulin only**^**(5)**^	705.6	43	6.09	1476.6	39	2.64
**Insulin plus metformin**	8853.8	293	3.31	5116.1	82	1.60
**Insulin plus insulin sensitizer**^**(6)**^	10271.5	379	3.69	5991.6	111	1.85
**Insulin plus secretagogue**^**(7)**^	6747.5	226	3.35	3609.0	59	1.63

^(1)^ participant reported a hypoglycemic event while taking the medication.

^(2)^ any combination of oral medications but no insulin.

^(3)^ metformin + TZD, regardless of other medications.

^(4)^ metformin + TZD + sulfonylurea, regardless of other medications.

^(5)^ any combination of insulin but no oral medications.

^(6)^ insulin sensitizer = TZD, metformin.

^(7)^ insulin secretogogues = sulfonylurea, meglitinides (prandin), incretins (exenatide).

Determination of the risk of experiencing a hypoglycemic event by different categorizations of medications (see Table 4) revealed a lower risk of hypoglycemia among those treated with oral agents only compared to those treated with insulin only (INT adjHR 0.53, 95%CI 0.36-0.78; STD adjHR 0.13, 95%CI 0.08-0.24). No difference was seen among those treated with oral agent and insulin compared to insulin alone. Within the intensive glycemia participants, few differences were seen with the addition of a sensitizer to insulin, or sulfonylurea to metformin and TZD use; however, within standard glycemia participants, those using insulin alone had a higher rate of HMA than those prescribed no sensitizer or only a sensitizer. In the intensive group, there was no difference in risk of hypoglycemia by number of medications while a higher risk with 3 or more medications was seen in the standard group.

**Table 4:** **Adjusted hazard ratio (HR) of severe hypoglycemia by combination of classes**^**(1)**^

**Medication Use in Interval Antecedent to Ascertainment of Event Status**	**Intensive**			**Standard**		
	**HR**	**95% CI**	**P value**	**HR**	**95% CI**	**P value**
Just Oral Agents vs. Just Insulin	0.53	(0.36, 0.78)	<.0001	0.13	(0.08, 0.24)	<.0001
Oral Agents plus Insulin vs. Just Insulin	0.93	(0.67, 1.31)	0.69	0.97	(0.66, 1.43)	0.89
No Glycemia Medication^(2)^ vs. Just Insulin	1.47	(0.35, 6.1)	0.60	0.15	(0.02, 1.09)	0.06
Insulin plus Sensitizer vs. Just Insulin^(3)^	1.19	(0.88, 1.61)	0.25	0.98	(0.69, 1.40)	0.93
No Insulin or Sensitizer^(4)^ vs. Just Insulin^(3)^	1.32	(0.56, 3.11)	0.52	0.22	(0.08, 0.62)	<.0001
Just Sensitizer vs. Just Insulin^(3)^	0.66	(0.45, 0.94)	0.02	0.14	(0.07, 0.25)	<.0001
Metformin + Sulfonlyurea + TZD vs. Metformin + TZD^(5)^	0.94	(0.72, 1.23)	0.65	0.60	(0.32, 1.11)	0.10
2 meds vs. 0 or 1 meds	0.83	(0.46, 1.5)	0.53	1.08	(0.64, 1.82)	0.78
3 meds vs. 0 or 1 meds	1.04	(0.59, 1.82)	0.89	1.82	(1.10, 3.00)	0.02

^(1)^ All results control for the following baseline covariates: age, gender, race, education, time since diabetes diagnosis, history of neuropathy/nerve problems, BMI, A1C, albumin to creatinine ratio, serum creatinine, LDL-C, and factors used to stratify randomization (treatment groups within the BP and Lipid trials and the presence of clinical cardiovascular disease)

^(2)^ In the standard group, there were 430 person years out of a total of 17,698 person years (2.4% of all follow-up) and in the intensive group, 35 person years out of a total of 17,130 person years (0.2%) in which participants were on no glycemia medications.

^(3)^ Model also controls for use of AGI’s, meglitinide, incretins and sulfonylurea.

^(4)^ Participants in this group were prescribed sulfonylureas, AGI, meglitinide, incretin or exenatide.

^(5)^ Model also controls for use of Insulin, AGI’s, meglitinide, incretins and sulfonylurea.

Determination of the hazard ratio for use of each class of medication (see Tables 5 and 6), found that the lowest risk was for those given a prescription of a biguanide compared to those that were not prescribed this medication (INT adjHR 0.78, 95%CI 0.63-0.96; STD adjHR 0.73 95%CI 0.53-1.00) and that the highest risk was among those prescribed a TZD (INT adjHR 1.62, 95%CI 1.33-1.99; STD adjHR 1.83 95%CI 1.35-2.48) and those prescribed insulin (see Tables 5 and 6 for hazard ratios). Further exploration of the increased risk among those prescribed a TZD found that the highest rate of events was among those participants treated with insulin plus a TZD but not with a sulfonylurea plus a TZD in both the intensive group (4.48 events per 100 person-years) and the standard group (3.42 events per 100 person-years) (see appendix material).

**Table 5:** **Unadjusted and adjusted HR of severe hypoglycemia by medication class - Intensive group participants**^**(1)**^

**Medication Use in Interval Antecedent to Ascertainment of Event Status**	**Unadjusted**			**Adjusted**^**(2)**^		
	**HR**	**95% CI**	**P value**	**HR**	**95% CI**	**P value**
Sulfonylurea	0.87	(0.73, 1.04)	0.12	1.06	(0.88, 1.28)	0.54
Biguanide	0.73	(0.59, 0.89)	<.0001	0.78	(0.63, 0.96)	0.02
TZDs	1.40	(1.15, 1.70)	<.0001	1.62	(1.33, 1.99)	<.0001
AGIs	1.05	(0.71, 1.54)	0.81	1.10	(0.74, 1.62)	0.64
Meglitinide	0.91	(0.74, 1.12)	0.37	1.10	(0.88, 1.38)	0.38
Incretin	0.97	(0.51, 1.82)	0.92	1.06	(0.56, 1.99)	0.86
Bolus Insulin	1.62	(1.35, 1.95)	<.0001	1.58	(1.26, 1.98)	<.0001
Basal Insulin	1.61	(1.30, 1.99)	<.0001	1.44	(1.13, 1.85)	<.0001
Pre-Mixed Insulin	1.41	(0.94, 2.13)	0.10	1.91	(1.25, 2.92)	<.0001

**Table 6:** **Unadjusted and adjusted HR of severe hypoglycemia by medication class - Standard group participants**^**(1)**^

**Medication Use in Interval Antecedent to Ascertainment of Event Status**	**Unadjusted**			**Adjusted**^**(2)**^		
	**HR**	**95% CI**	**P value**	**HR**	**95% CI**	**P value**
Sulfonylurea	0.51	(0.37, 0.69)	<.0001	0.85	(0.6, 1.20)	0.36
Biguanide	0.54	(0.40, 0.74)	<.0001	0.73	(0.53, 1.00)	0.05
TZDs	1.47	(1.09, 1.98)	0.01	1.83	(1.35, 2.48)	<.0001
AGIs	1.48	(0.54, 4.01)	0.44	1.45	(0.52, 4.03)	0.48
Meglitinide	1.34	(0.85, 2.10)	0.21	1.50	(0.93, 2.42)	0.10
Incretin	1.84	(0.58, 5.86)	0.30	1.80	(0.56, 5.74)	0.32
Bolus Insulin	2.25	(1.63, 3.09)	<.0001	1.68	(1.14, 2.47)	0.01
Basal Insulin	2.72	(1.95, 3.79)	<.0001	0.85	(0.60, 1.20)	0.36
Pre-Mixed Insulin	2.24	(1.56, 3.23)	<.0001	0.73	(0.53, 1.00)	0.05

^(1)^ Hazard ratio for use of medication as part of glycemia medication regimen compared to participants who were not prescribed medication. All results control for the following baseline covariates: age, gender, race, education, time since diabetes diagnosis, history of neuropathy/nerve problems, BMI, A1C, albumin to creatinine ratio, serum creatinine, LDL-C, and factors used to stratify randomization (treatment groups within the BP and Lipid trials and the presence of clinical cardiovascular disease).

^(2)^ Adjusted for baseline covariates and other listed glycemia medications.

## Discussion

Our analysis of hypoglycemic events in the ACCORD trial shows that a large portion of events are preceded by behavioral-related situations that are potentially modifiable or preventable. Over half of all hypoglycemia episodes in both the standard and intensive group were related to either delaying or missing a meal or eating less food than normal. Additional behaviors that preceded hypoglycemic events included increased physical activity and incorrect medication use although incorrect medication use was listed as an antecedent (1-8% depending on medication) much less frequently than diet related behaviors. At the time of reporting severe hypoglycemic events, participants and staff were also asked about changes in medications that were not used to treat diabetes but known to either lower blood glucose or mask the symptoms of low blood glucose (e.g. beta blockers or ACE inhibitors). While there was differential reporting of these as antecedents between the intensive interventional study group (2%) and the standard interventional study group (8%), overall, this was infrequently listed as an antecedent in comparison to behavioral factors.

The consequences of severe hypoglycemia were frequently clinically significant. A large number of the hypoglycemic events reported in this paper resulted in hospitalization. This is in part due to the definition of severe hypoglycemia used in the ACCORD study (low blood glucose and treatment by medical personnel). Importantly, approximately 25% of the severe hypoglycemic incidents reported by the ACCORD participants resulted in loss of consciousness, about a third in confusion/irrational behavior, and 6% resulted in a personal accident or injury. Also of note, and perhaps associated with the more severe complications, the frequency of the neuroglycopenic symptoms of confusion/disorientation was equal to the adrenergic sweaty and shaky symptoms [12]. This combination of potentially dangerous consequences combined with the high frequency of preventable antecedents illustrates the importance of appropriate education of patients with diabetes on techniques to prevent hypoglycemia.

More events occurred among those who were prescribed insulin. This finding has been reported in other papers [13,14]. This may be due to the action of insulin or due to the clinical characteristics of participants for whom insulin is prescribed or both. ACCORD was a clinical trial testing strategies for glycemic control and a variety of medications were available for use to obtain the target A1Cs in each arm of the trial. The recommended study algorithm of treatments suggested the use of oral medications before addition of insulin. Thus, participants who were prescribed insulin may have represented those participants whose disease was more difficult to control. They may also represent participants who had contraindications, such as impaired renal function, to some oral agents.

As expected, we found a low rate of severe hypoglycemic events associated with the biguanide class. This has also been reported in other papers [13,15]. In contrast, we unexpectedly found high rates of hypoglycemia among those participants treated with TZD. Few participants were prescribed TZDs alone so we cannot determine the rate among those who used this medication alone. When we examined the use of TZD in conjunction with sulfonylureas and insulin (two of the most commonly used medications in ACCORD), we found the highest rates of hypoglycemia among those who were prescribed TZDs in combination with insulin as compared to the combination of TZD with sulfonylureas. Among those participants who were prescribed TZD but not sulfonylurea or insulin, the incidence rate of severe hypoglycemia was similar to that reported in a prescription monitoring program [16]. Although the mechanism for this finding is not clear, it is possible that the insulin sensitizing action of the TZD accentuates the risk of hypoglycemia of insulin when these two are used in combination. Alternatively, rosiglitizone has been shown to preserve [17] and possibly improve pancreatic beta cells function [18]. This preservation/improvement in the responsiveness of the beta cells in combination with exogenous insulin and improved insulin sensitivity could lead to increased risk of hypoglycemia.

There are several strengths to this analysis. We collected a large amount of data about symptoms, antecedents, and consequences on all severe hypoglycemic events. However, we did not obtain similar information in those participants that did not experience hypoglycemia and cannot determine the risk of hypoglycemia with the various antecedents. ACCORD had a large study population and used a number of common diabetes medications allowing us to examine the rate of hypoglycemia in a variety of medications and combinations. However, the study tested strategies, and participants were not randomly assigned to particular medications. Thus, this analysis can only provide associations. We focused on first events in this paper; when we conducted similar analysis on symptoms, antecedents and consequences of the second reported severe hypoglycemic event, we found no significant difference between study arms.

There are some limitations that should be discussed. There is considerable confounding between participant characteristics and the prescription of certain medications. While we have attempted to account for this in our analysis, it is likely there is still some residual confounding. ACCORD did not capture actual medication use. This analysis instead utilizes prescription of medication. It is possible that some medications prescribed were not actually taken by the study participant. Symptoms, antecedents and consequences of the hypoglycemic events rely to some degree on patient self-report and thus may be subject to under or over-reporting.

## Conclusion

What potential beneficial actions might these data suggest? Confusion and loss of consciousness was often seen as consequences of severe hypoglycemia. These serious outcomes suggest the need for clinicians to help their patients develop a proactive plan for prevention which includes recognition of symptoms such as confusion or disorientation as potential signs of hypoglycemia. While ACCORD was not designed to evaluate preventative measures, we did find lower overall rates of hypoglycemia in the standard group. The more frequent use of oral medications in the standard group suggests that these medications should be considered in those experiencing severe hypoglycemia. The biguanide class of medication had the lowest risk of hypoglycemia; adjustment of medication toward biguanides where possible is an option to consider.

Perhaps the most important finding from these analyses is that over half of hypoglycemic episodes were preceded by a decrease in carbohydrate intake. With proactive planning by patients with diabetes, this behavior could be changed with a potentially large impact on the risk of severe hypoglycemia. Adding these potential tools into already existing algorithms for preventing severe hypoglycemic episodes, could provide the patient and clinician more choices for shared decision making to avoid severe events.

## Competing interests

The authors declare the following conflict of interest:

Bonds, Miller, Dudl, Feinglos, Malozowski, Seaquist, Simmons: No Conflicts of Interest

Ismail-Beigi: Consultant, Eli Lilly Corporation

Sood: Consultancies: Pfizer, Abbott, Medtronic (All one time only, 2005–2008), Honoraria for public speaking: Pfizer, Novartis (All one time only, 2006)

## Authors’ contributions

DEB researched the data, wrote the paper; MEM conducted the statistical analysis, wrote sections of the paper, edited the manuscript; MF researched the data, edited the manuscript, contributed to the discussion; FIB researched the data, edited the manuscript, contributed to the discussion; SM edited the manuscript, contributed to the discussion; ES researched the data, edited the manuscript, contributed to the discussion; DLS researched the data, edited the manuscript, contributed to the discussion; AS researched the data, edited the manuscript, contributed to the discussion. All authors read and approved the final manuscript.

## Pre-publication history

The pre-publication history for this paper can be accessed here:

http://www.biomedcentral.com/1472-6823/12/5/prepub
